# Evolutionary dynamics of complex multiple games

**DOI:** 10.1098/rspb.2019.0900

**Published:** 2019-06-26

**Authors:** Vandana Revathi Venkateswaran, Chaitanya S. Gokhale

**Affiliations:** Research Group for Theoretical Models of Eco-evolutionary Dynamics, Department of Evolutionary Theory, Max Planck Institute for Evolutionary Biology, August Thienemann Strasse 2, 24306 Plön, Germany

**Keywords:** evolutionary game theory, multiplayer games, multiple games, finite population

## Abstract

Evolutionary game theory has been successful in describing phenomena from bacterial population dynamics to the evolution of social behaviour. However, it has typically focused on a single game describing the interactions between individuals. Organisms are simultaneously involved in many intraspecies and interspecies interactions. Therefore, there is a need to move from single games to multiple games. However, these interactions in nature involve many players. Shifting from 2-player games to multiple multiplayer games yield richer dynamics closer to natural settings. Such a complete picture of multiple game dynamics (MGD), where multiple players are involved, was lacking. For multiple multiplayer games—where each game could have an arbitrary finite number of players and strategies, we provide a replicator equation for MGD having many players and strategies. We show that if the individual games involved have more than two strategies, then the combined dynamics cannot be understood by looking only at individual games. Expected dynamics from single games is no longer valid, and trajectories can possess different limiting behaviour. In the case of finite populations, we formulate and calculate an essential and useful stochastic property, fixation probability. Our results highlight that studying a set of interactions defined by a single game can be misleading if we do not take the broader setting of the interactions into account. Through our results and analysis, we thus discuss and advocate the development of evolutionary game(s) theory, which will help us disentangle the complexity of multiple interactions.

## 1. Introduction

Evolutionary game theory [1–4] has been used to study phenomena ranging from the dynamics of bacterial populations to the evolution of social behaviour. In evolutionary games, individuals are cast as players that interact with each other in ‘games’, which are metaphorical summaries of interactions. For example, in the classical Prisoners’ dilemma, individuals can either cooperate or defect, and each pairwise interaction results in a payoff for the players involved [3,5]. Over time, players adopt a strategy which either performs better or worse than the average of the population and thus increases or decreases in frequency. Tracking the change in their frequencies over time, evolutionary dynamics can provide insight into the eventual fate of the strategies in a game, e.g. whether they dominate, coexist, or go extinct from the population [3].

**Figure 1.**
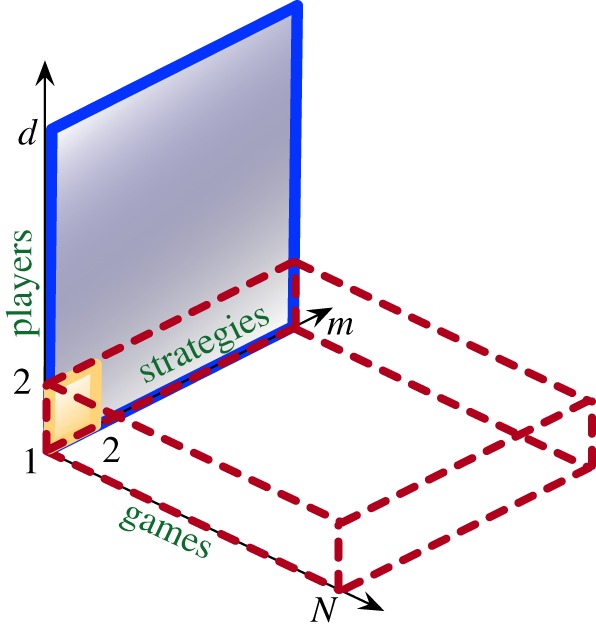
Scope of this study. Typical evolutionary game dynamics focuses on 2-player games with two strategies (solid yellow square). Extensions to multiplayer games (*d*) and multiple strategies (*m*, solid blue rectangle) expands the domain of study to public goods games and other social dilemmas. However, this is still limited to a single game. Hashimoto [8] has extended 2-player multi-strategy games in a novel direction of multiple games (*N*, dotted red cuboid). Our work generalizes this approach and develops a method for analysing multiple games, where each involved game could be a multiplayer (and multi-strategy) game. Thus, this approach enables us to study the entire space of multiple games (*N*) with multiple strategies (*m*) consisting of multiple players (*d*). (Online version in colour.)

Considerable effort has gone into making games more realistic (with interactions among multiple players and allowing players to adopt strategies from a large set [6,7]) shown by the solid blue rectangle in figure 1. As an example from the micro-scale, we discuss the interactions between microorganisms. One bacterium interacts with its neighbours. Assuming that a bacterium would interact only in a pairwise fashion is clearly an assumption. When more players are involved, dynamics can change not just quantitatively but qualitatively [9–11]. Multiplayer games in bacterial dynamics can better explain the coexistence of avirulent ‘cheaters’ and virulent ‘cooperators’ in populations of the pathogen *Salmonella* Typhimurium [12]. Likewise, in *Pseudomonas fluorescens* communities, the seemingly destructive cheating cells can promote evolution of collectives [13], an inherently multiplayer interaction. The dynamics between the microbes constituting the microbiome are nonlinear, lending themselves to multiplayer games [14]. A constituent of the microbiome may not be playing a single multiplayer game with the other constituents but is also interacting with the host. The complete interaction in the holobiont would then be a collection of several multiplayer games [15].

Do we consider all the different games singly or as one massive game with a large number of complex strategies? The answer in short is that under certain conditions, the single games studied individually do not provide the same results as when we infer single games from the combined dynamics. Across scales of organization, single games fail to satisfactorily capture dynamics ranging from bacterial dynamics (as above) to human behaviour. Envision the interactions in public goods games such as climate change issues [16]. When nations’ leaders discuss strategies to improve the global climate status, they also need to take into account the interests of the people they are representing. If the leaders agree to contribute towards achieving the goals of the climate summit, it often comes at a cost to the private interests of the nation. Using a different set of strategies, the leaders have to then appease the electorate. Thus, political leaders are playing at least two multiplayer games: one with other nations and another within their nation. Therefore, we need to shift from single game dynamics (SGD) to multiple game dynamics (MGD) as shown by the dotted red cuboid in figure 1. Previous studies on MGD have shown that a combination of games with more than two strategies is inseparable into its constituent SGD [8]. However, this result is valid only for 2-player games as shown in the figure. It ignores the complexity of multiplayer games as discussed above. We have developed a method for analysing multiplayer MGD.

Besides ecological examples, formal analysis of evolutionary games in finite populations implies the role of multiple games. The assumption of weak selection, where the game has a weak effect on an organism’s fitness, typically is done not only for mathematical ease but also assuming that, the payoff differences are small, the strategies are similar, or the individuals are confused about the strategies [17]. Multiple games provide a simpler alternative where each game has a small effect on an individual's fitness.

A complete picture of MGD, where multiple players are involved, is lacking. Nonlinearity in the replicator dynamics increases with increasing number of players. As a result, multiplayer games can have multiple internal equilibria as opposed to 2-player games that have at most one internal equilibrium solution [10]. An initial condition within the MGD space can converge to another equilibrium solution than expected from the SGD. Thus, if we are aware that the dynamics are composed of a set of different games, then is the simplified use of a single bigger game justified? In other words, can the MGD be decomposed into its constituent SGDs? If yes—the conclusions drawn from individual games are valid. If not—it will be necessary to use MGD to obtain realistic results.

To answer this question, we first present a complete and general method to study multiple games with many strategies and players, all at once (figure 1). When the games have more than two strategies, we find that the MGD do not correspond to the dynamics of its constituent single games, in line with previous findings, while we also extend the analysis to finite populations. Then we discuss a specific model on how the inclusion of two different games (territorial defence and hunting) can result in the observed division of labour in lionesses [18,19]. Further, we show that for some initial conditions the MGDs and SGDs differ not only in the dynamics but the resulting equilibria as well.

## 2. Model

### (a) Single game dynamics

2-player games with two strategies have been studied extensively, both in infinite as well as finite populations. A game between two individuals can be represented by the following payoff matrix:2.1
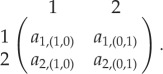
The matrix represents a symmetric 2-strategy 2-player game. We do not study asymmetric games [20]. The two individuals, focal and co-player are represented by a row and a column, respectively. Each player adopts one of the two strategies, 1 or 2. We write the elements of the matrix in the form *a*_*i*,*α*_, where *i* is the strategy of the focal (or row) player. The vector *α* is written as *α* = (*α*_1_, *α*_2_) where *α*_*i*_ indicates the number of strategy *i* individuals the focal individual interacts with. For example, in a 3-player game with two strategies, the payoff entry *a*_2,(1,1)_ corresponds to a focal player with strategy 2 interacting with two other players with strategies 1 and 2, respectively.

The average payoff obtained from the game is the reproductive success of that strategy [21]. This analysis has been extended to interactions having *multiple* strategies [22] as well as *multiple* players [23,24]. To make our notation clear, we illustrate a payoff matrix for a multiplayer (*d* player) game with two strategies as


2.2
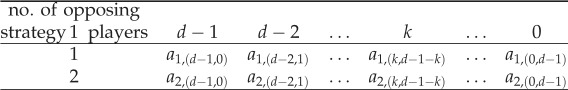


Even when extending the number of strategies, the dynamics of this complicated system can still be analysed by the replicator dynamics [25,26]. For a *d* player game with *m* strategies, the replicator dynamics is given by a set of *m* differential equations: ${\dot{x}}_{i}=x_{i}(f_{i}-\phi )$ where *x*_*i*_ is the frequency of strategy *i*, and *f*_*i*_ is the average payoff of the strategy *i*. The average payoff of the population is given by $\phi =\sum_{\;j=1}^{m}x_{j}f_{j}$. This simple evolutionary game framework has been used to describe a wide range of phenomena from chemical reactions of prebiotic elements to the evolution of social systems [27].

While this extension to multiple players and strategies is not trivially obtained [28], it still belongs to the domain of a single game. The framework lacks the ability to incorporate interactions which have differential impacts on fitness. Therefore, we now incorporate multiple games and measure their cumulative impact on individual fitness.

### (b) Multi-game dynamics

**Figure 2.**
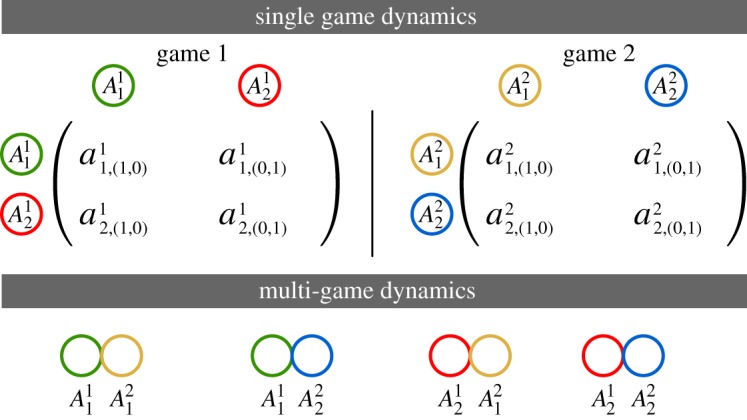
From SGD to MGD. The population after combination is divided into four types: playing strategy 1 in game *A*^1^ and game *A*^2^, strategy 1 in *A*^1^ and 2 in *A*^2^, strategy 2 in *A*^1^ and 1 in *A*^2^. And finally, strategy 2 in *A*^1^ and *A*^2^. Thus, we have four types of strategies, $A_{1}^{1}A_{1}^{2}$, $A_{1}^{1}A_{2}^{2}$, $A_{2}^{1}A_{1}^{2}$, and $A_{2}^{1}A_{2}^{2}$. Their respective frequencies are *x*_11_, *x*_12_, *x*_21_, and *x*_22_. Since there are four ‘categorical types’, the dynamics is shown in an *S*_4_ simplex. (Online version in colour.)

Individuals may employ different strategies in various games (e.g. division of labour scenarios [29]) and their (average) payoffs will depend on their performance in all such games. Switching between such socially driven games is realistic and not only a matter of theoretical interest but has been experimentally explored as well [30]. This section generalizes the multi-game approach by Hashimoto [8] to an arbitrary number of players. To contrast MGD with the previously discussed SGD, consider a simple example of two, 2-player games, each having two strategies:
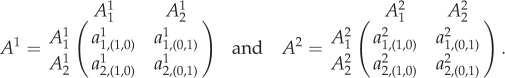
Combining the strategies from the above two games results in four categories of individuals. The frequencies of the four categories are given by *x*_11_, *x*_12_, *x*_21_, and *x*_22_ where the first and second positions (in the subscript) denote the strategies adopted in games 1 and 2, respectively (figure 2).

For a combination of *N* games, each game *j* can be described by a payoff matrix *A*^*j*^. Each game *j* could be a *d*_*j*_ player game with *m*_*j*_ number of strategies. The categorical frequencies would then be given by $x_{i_{1}i_{2}\ldots i_{j}\ldots i_{N}}$, where *i*_*j*_ is the strategy being played in game *j*. The frequencies of the individual strategies for all *N* games can be written down as

2.3 $$p_{\;ji_{j}}=\sum_{k=1,k\neq j}^{k=N}\sum_{i_{k}=1}^{m_{k}}x_{i_{1}i_{2}\ldots i_{j}\ldots i_{N}},$$

which allows us to compute the fitness of strategy *i*_*j*_ as

2.4 f jij=∑|α|=dj−1(dj−1α)pαaij,α j.

As before, $\alpha_{m_{j}}$ is the number of strategy *m*_*j*_ players. Using multi-index notation, we have $\alpha =(\alpha_{1},\alpha_{2},\ldots ,\alpha_{m_{j}})$ which gives us the multinomial coefficient, with the absolute value $|\alpha |=\alpha_{1}+\alpha_{2}+\cdots +\alpha_{m_{j}}$ and the power $p^{\alpha }=p_{\;j1}^{\alpha_{1}}p_{\;j2}^{\alpha_{2}}\ldots p_{\;jm_{j}}^{\alpha_{m_{j}}}$. The average fitness of the population is given by, *ϕ*_*j*_ = (**pf**)_*j*_. Using this, we can write down the time evolution of all the categorical strategies as

2.5 $${\dot{x}}_{i_{1}i_{2}\ldots i_{j}\ldots i_{N}}=x_{i_{1}i_{2}\ldots i_{j}\ldots i_{N}}\left(\sum_{\;j=1}^{N}(f_{\;ji_{j}}-\phi_{j})\right).$$

This system of equations is reminiscent of the replicator equation for the SGD. The summation in the MGD replicator equations is due to an assumption of additive fitness effects from all games [8]. In the following sections, we will explore the use of this formulation for multiple games where each game can have a different number of players. Through the examples of specific cases, we aim to highlight the general principles of multiple games.

## 3. Results

### (a) Multiplayer game(s) with multiple strategies

Combining multiplayer games, frequency feedback between strategies is possible. Moreover, an individual can take part in different interactions. A lioness can be part of forming the defensive line (tragedy of the commons) and hunting (stag–hunt game). Strategies in game 1 would be *Cooperator*, *Defector*, *Loner*, etc. Strategies in game 2 could be hunting positions *Wing,* *Centre*, and so on. Thus in our framework, an individual can have utterly different strategy sets for each game.

#### (i) 2-player game with 2-strategies+3-player game with 2-strategies.

To illustrate games with two strategies, we shall use the payoff matrices shown in (3.1).3.1
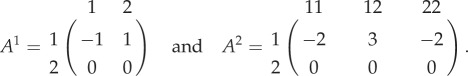
Here, *A*^1^ is a 2-player coexistence game and *A*^2^ is a 3-player game. In *A*^2^, the values *a*_1,(*k*,*d*−1−*k*)_ − *a*_2,(*k*,*d*−1−*k*)_ and *a*_1,(*k*+1,*d*−*k*)_ − *a*_2,(*k*+1,*d*−*k*)_ have different signs for all *k*. Thus solving for this scenario using our replicator-like equation (2.5), we have two interior fixed point solutions: a stable and an unstable. The equilibrium solutions for strategy 1 in the two SGDs in (3.1) are $q_{1}^{*}=0.5$ for *A*^1^ and $q_{2}^{*}=(q_{2_{1}}^{*},q_{2_{2}}^{*})=(0.27,0.73)$ for *A*^2^. Since *A*^2^ is a 3-player game, it has at most two internal equilibrium solutions [10]. The result of combining these games, i.e. their MGD, is shown in electronic supplementary material, figure A.4. The first panel shows the SGD of both the games *A*^1^ and *A*^2^. We choose three initial conditions *ic*_1_, *ic*_2_, and *ic*_3_ to understand the difference between SGD and MGD by following those trajectories’ dynamics in the SGDs and MGD. After combining the two games with two strategies, we obtain the MGD that has four (categorical) strategies *x*_11_, *x*_12_, *x*_21_, and *x*_22_. The dynamics are plotted in a three-dimensional simplex. All trajectories that start above the unstable equilibrium in *A*^2^ end up in the line given by *E*, the evolutionarily stable (ES) set. As shown in the third panel of electronic supplementary material, figure A.4, one can recover the SGD back from their combined game dynamics to compare the MGD and SGDs, i.e. re-obtain *p*_11_ (= *x*_11_ + *x*_12_), *p*_12_ (= *x*_21_ + *x*_22_), *p*_21_ (= *x*_11_ + *x*_21_), and *p*_22_ (= *x*_12_ + *x*_22_). As shown by the dynamics in this figure, the MGD is the same as the separate SGDs. So the MGD can be separated back into its constituent games when both games have two strategies.

#### (ii) 2-player game with 3-strategies+3-player game with 2-strategies.

**Figure 3.**
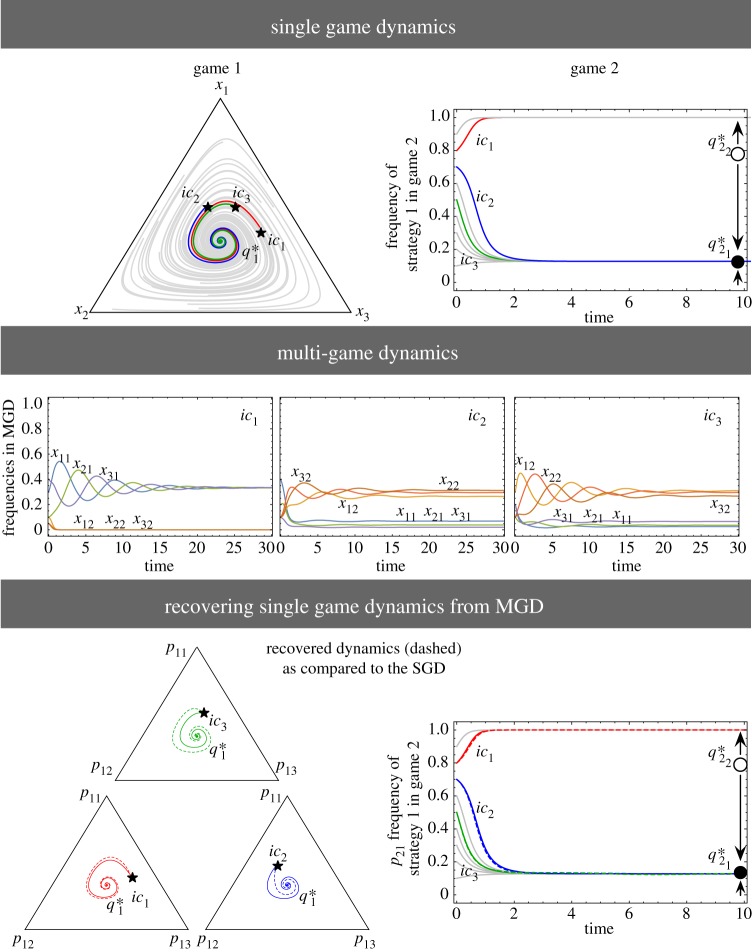
Two games, each having three and two strategies, respectively. The first row shows the SGD of the games in (3.2). Game 1 is a Rock–Paper–Scissor game with a stable internal equilibrium, $q_{1}^{*}=(1/3,1/3,1/3)$. Game 2 has two internal fixed points at $q_{2_{1}}^{*}=0.127$ (stable) and $q_{2_{2}}^{*}=0.740$ (unstable). The asterisks denote the positions from where the three trajectories *ic*_1_, *ic*_2_, and *ic*_3_ begin (initial conditions). The grey trajectories are other random initial conditions. For the MGD, we have six ‘categorical types’ *x*_11_, *x*_12_, *x*_21_, *x*_22_, *x*_31_, and *x*_32_. We plot the time evolution of the strategies for the three different initial conditions. From this MGD, we can recover the corresponding frequencies for the two SGD. These are plotted in the last row. The recovered *p*_11_ refers to playing strategy 1 in game 1, *p*_21_ refers to playing strategy 1 in game 2 and so on. All recovered trajectories (dashed) go to the same equilibria of the SGD in game *A*^1^ and in game *A*^2^ (solid). While the equilibria of the MGD correspond to that of the SGD, the dynamics can follow different routes. The initial conditions used for (*x*_11_, *x*_12_, *x*_21_, *x*_22_, *x*_31_, *x*_32_) are: *ic*_1_ = (0.3, 0.1, 0.1, 0.05, 0.4, 0.05), *ic*_2_ = (0.4, 0.1, 0.2, 0.1, 0.1, 0.1), and *ic*_3_ = (0.2, 0.3, 0.1, 0.1, 0.2, 0.1). (Online version in colour.)

Next, we increase the number of strategies in the 2-player game:3.2
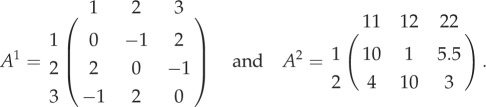
Now *A*^1^ is a Rock–Paper–Scissor game. Trajectories starting from any internal initial conditions converge to a unique stable equilibrium, $q_{1}^{*}=(1/3,1/3,1/3)$ [3]. For *A*^2^, the equilibrium solutions are $q_{2_{1}}^{*}=0.127$ (stable) and $q_{2_{2}}^{*}=0.740$ (unstable). The MGD takes place in a six-dimensional space, thus to compare the MGD with their SGDs we project them in the SGD space as shown in figure 3. The SGD for *A*^1^ and *A*^2^ are shown in the first panel. Since the two games, *A*^1^ and *A*^2^ have three and two strategies; respectively, their combined MGD will have six categorical strategies. The bottom panel displays the plots that compare the SGDs recovered from the MGD (dashed lines) with the original SGDs (solid lines). The recovered dynamics do not match that of the individual games. Thus, increasing the number of strategies in at least one game shows that the MGD differs from the SGDs. Therefore, while modelling multiplayer game scenarios with more than three strategies that involve individuals participating in multiple interactions simultaneously, one must look at their combined game dynamics to study the full picture [8]. We extend the domain of such multiplayer, multiple games analysis where both games have three strategies in the next section.

#### (iii) 2-player game with 3-strategies+4-player game with 3-strategies.

**Figure 4.**
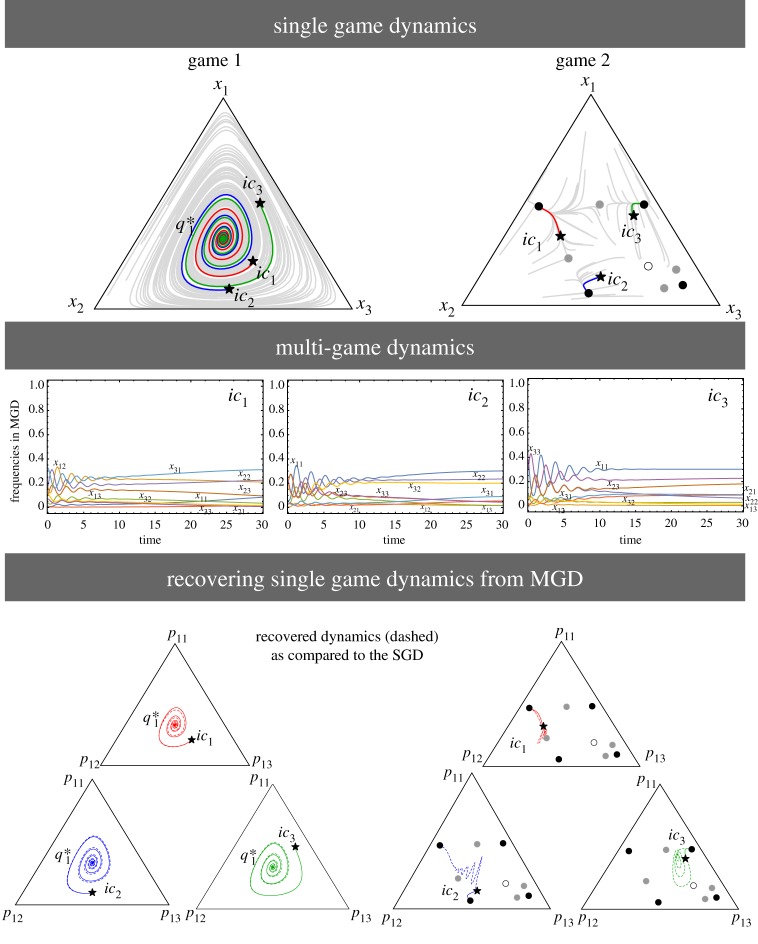
Two games having three strategies. In the SGDs of the individual games, *A*^1^ has a stable equilibrium solution $q_{1}^{*}=(1/3,1/3,1/3)$ and *A*^2^ has in total nine interior equilibrium solutions: four stable (dark circles), one unstable (open circle), and four saddle points (grey circles). The asterisks in the triangular *S*_3_ simplex denote the initial conditions (*ic*_1_, *ic*_2_, and *ic*_3_) of interest, whereas the grey trajectories are other random initial conditions. When both games contain three strategies, nine categorical types are possible. For visualizing the MGD, we show the time evolution of the nine strategies. Retrieving the distribution of frequencies of strategies in the SGDs from the MGD, for *A*^1^ again, while the equilibrium values are all the same $q_{1}^{*}$, the dynamics are different. However, for *A*^2^, *ic*_1_ and *ic*_3_ end up in the same equilibria in the MGD as in their respective SGDs, *ic*_2_ changes equilibrium. The initials conditions used for (*x*_11_, *x*_12_, *x*_13_, *x*_21_, *x*_22_, *x*_23_, *x*_31_, *x*_32_, *x*_33_) are : *ic*_1_ = (0.01, 0.166, 0.038, 0.002, 0.176, 0.102, 0.3251, 0.111, 0.070), *ic*_2_ = (0.058, 0.005, 0.029, 0.027, 0.205, 0.212, 0.050, 0.190, 0.224), and *ic*_3_ = (0.176, 0.066, 0.024, 0.002, 0.176, 0.002, 0.225, 0.111, 0.218). (Online version in colour.)

Finally, we illustrate a case of having three strategies in both games (shown in matrices (3.3)). *A*^1^ is a Rock–Paper–Scissor game like the one discussed in the previous example. *A*^2^ is a 4-player 3-strategy game used previously in [10]. In the SGDs of the individual games, *A*^1^ has a stable equilibrium $q_{1}^{*}=(1/3,1/3,1/3)$ and *A*^2^ has in total nine interior equilibrium solutions: four stable, one unstable, and four saddle points. The SGDs of *A*^1^ and *A*^2^ and their MGD are shown in figure 4.


3.3
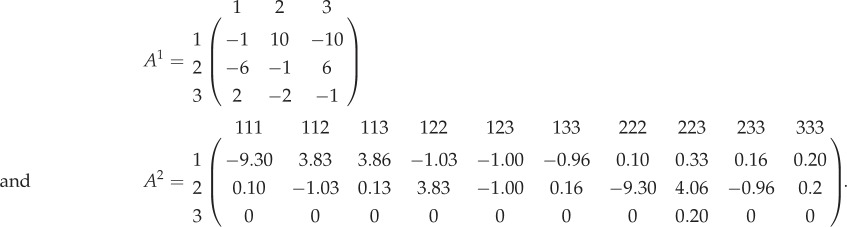


The results show that in the MGD it is even possible for an initial condition to end up in a completely different equilibrium as opposed to the SGD.

Consider *A*^2^ which has four stable internal equilibria. In figure 4 top row, the three initial conditions go to three of the stable equilibria. After combining with *A*^1^ and then recovering the dynamics of *A*^2^, we see that *ic*_2_ switches its long-term equilibrium behaviour (figure 4 bottom row, recovered dynamics). Multiplayer games offer the possibility of multiple internal equilibria and combined games can allow the trajectories to switch between them. Thus, the constituent games of an MGD, especially involving multiplayer games should be studied with scrutiny since their long-term evolutionary trajectory cannot be predicted by the basins of attractions of the SGD.

In a previous study of 2-player games with two strategies [31], it was shown that the SGD can be obtained back from their MGD. The dynamics lie on the generalized invariant *manifold* [25,32] in the *S*_4_ simplex which is given by *W*_*K*_ = {*x* ∈ *S*_4_| *x*_11_ *x*_22_ = *K x*_12_*x*_21_} for *K* > 0. When *K* = 1, we have *W* = {*x* ∈ *S*_4_| *x*_11_ *x*_22_ = *x*_12_*x*_21_} which is the *Wright manifold*. On this manifold, MGD can be separated back into the SGDs of the constituent games (see the electronic supplementary material for details). The attractor for a combination of two 2-player games having two strategies each is a line *E*, an ES set [31]. The point where the line *E* intersects the Wright manifold indicates a rest point. All the trajectories in the simplex depicting the MGD fall onto an attractor given by a line (ES set) on *W*_*K*_. The dynamics on *W*_*K*_ and the trajectories on each *W*_*K*_ were analysed in the same study [31] and the conditions when they are qualitatively the same as on the Wright manifold. However, for multiple games having more than two strategies in at least one game, the MGD cannot be separated even into a linear combination of the constituent SGDs unless they are on *W* [8]. Increasing the number of games and strategies increases the dimension of MGD simplex and also that of the Wright manifold. Only on the Wright manifold can the MGD be separated back into its SGDs (see the electronic supplementary material for details). Therefore, it is important to know on which manifold the initial conditions are, for only if they start from the Wright manifold *W*, will the dynamics be a perfect match to the SGDs [8].

Multiple multiplayer games can give rise to numerous rest points, and they can criss-cross with the Wright manifold which for multiple strategies would be of a dimension $\Sigma_{i=1}^{N}(m_{j}-1)$, where *N* is the number of games and *m*_*j*_ is the number of strategies in game *j* (see the electronic supplementary material). Future work on multiple *d*-player games with many strategies could involve finding traversable paths in this complex space as is shown by some unusual trajectories (figure 4). Differing from the earlier work on 2-player multiple games [8,31], we show that MGDs cannot always be trivially separated into their constituent SGDs in multiplayer games with multiple strategies. Furthermore, including multiplayer games in combined games can lead to the SGD and the recovered dynamics differing not just in the dynamics of trajectories but also in their eventual end points. We have a generalized method that looks at a combination of many multiplayer games having diverse strategy sets.

Until now, the analysis firmly rested on the deterministic dynamics and on the derivation and analysis of the replicator-like equation. This assumes an infinitely large population. To understand combined games in realistic finite populations, we turn our attention to stochastic methods.

### (b) Finite population

Evolutionary dynamics in finite populations has the potential of having qualitatively different dynamics than their deterministic analogues [33]. In finite populations, the size of the population controls the balance between selection and drift with smaller populations showing higher levels of stochasticity. We use a birth–death Moran process to model a finite population of size *Z* in our framework [33,34]. An individual is chosen (proportional to its fitness) to reproduce an identical offspring. Another individual is chosen randomly for death. Thus, the total population size remains constant. Earlier we assumed that the fitness of a strategy was its average payoff. Besides the population size, we can control the effect of the game on the fitness via a particular mapping of payoff to fitness. The mapping could be a linear function *f* = 1 − *w* + *wπ* where *w* is the selection intensity [3]. If *w* = 0, selection is neutral whereas for *w* = 1 selection is strong and the payoff determines the fitness completely. However, since negative fitnesses in this framework are meaningless, there are restrictions on the range of *w*. Alternatively, to avoid this restriction, we can use an exponential function *f* = e^*wπ*^ [35]. Under any mapping scenario but weak selection, the fixation probability of strategy 1 in a population of *Z* − 1 strategy 2 players playing a *d*-player game, is [10],

3.4 $$\rho_{1}\approx \frac{1}{Z}+\frac{w}{Z^{2}}\sum_{m=1}^{Z-1}\sum_{\gamma =1}^{m}(\pi_{1}-\pi_{2}),$$

where *π*_*i*_ is the fitness of strategy *i* and the payoffs depend on the number of mutants *γ*. We have generalized this result to multiple games. The strategies in a multiple game are categorical ones. For instance, a two game system with each game containing two strategies, has four categorical strategies as shown in figure 2. If one of the categorical strategies takes over the entire population, we term it as the fixation of the strategy defined by the category. If in a population of size *Z* playing *N* games, there is a single individual playing strategy $A_{i_{1}}^{1}A_{i_{2}}^{2}\ldots A_{i_{N}}^{N}$ in a population of *Z* − 1 individuals playing strategy $A_{h_{1}}^{1}A_{h_{2}}^{2}\ldots A_{h_{N}}^{N}$ then we are interested in the probability that this single individual takes over the population. First we need to map the payoffs to fitness and there are two ways of implementing any kind of mapping for multiple games: *Method I.* For each game, the payoffs are mapped to fitness and then the cumulative fitness is calculated. Here, the fixation probability of a single individual of type $A_{i_{1}}^{1}A_{i_{2}}^{2}\ldots A_{i_{N}}^{N}$ in a population of $A_{h_{1}}^{1}A_{h_{2}}^{2}\ldots A_{h_{N}}^{N}$ is given by (see the electronic supplementary material for details)

3.5 $$\begin{array}{r} \rho_{A_{i_{1}}^{1}A_{i_{2}}^{2}\ldots A_{i_{N}}^{N},\;A_{h_{1}}^{1}A_{h_{2}}^{2}\ldots A_{h_{N}}^{N}}\approx \frac{1}{Z} \end{array}+\frac{w}{NZ^{2}}\left[\sum_{m=1}^{Z-1}\sum_{\gamma =1}^{m}\left(\sum_{\;j=1}^{N}(\pi_{\;ji_{j}}-\pi_{\;jh_{j}})\right)\right].$$

*Method II.* The payoffs can be added first and then mapped to fitnesses. The fixation probability through this method is (see the electronic supplementary material for details)

3.6 $$\begin{array}{r} \rho_{A_{i_{1}}^{1}A_{i_{2}}^{2}\ldots A_{i_{N}}^{N},\;A_{h_{1}}^{1}A_{h_{2}}^{2}\ldots A_{h_{N}}^{N}}\approx \frac{1}{Z} \end{array}+\frac{w}{Z^{2}}\left[\sum_{m=1}^{Z-1}\sum_{\gamma =1}^{m}\left(\sum_{\;j=1}^{N}(\pi_{\;ji_{j}}-\pi_{\;jh_{j}})\right)\right].$$

For illustration, let us consider a combination of two games with two strategies each. For instance, the games in (3.1). We make pairwise comparisons between all categorical types, i.e. all the edges of the *S*_4_ simplex in electronic supplementary material, figure A.4. Using these comparative fixation probabilities, we can determine the flow of the dynamics over pure strategies. Let us focus on the edge $A_{1}^{1},A_{1}^{2}⇄A_{1}^{1},A_{2}^{2}$, where game 1 does not change and only game 2 matters. Hence, the fixation probabilities should be the same as if only game 2 exists. The single game fixation probability of game 2 is shown in electronic supplementary material, figure A.5. As given in equations (3.5) and (3.6), when game 2 is combined with game 1, there can be two ways of mapping payoffs to fitness. The results from these two methods in multiple games in finite populations are also plotted in electronic supplementary material, figure A.5.

The fixation probabilities of a strategy in a single game changes when ‘adding’ just one more game to it. Even on the edge $A_{1}^{1},A_{1}^{2}⇄A_{1}^{1},A_{2}^{2}$, where game 1 is neutral and only game 2 matters, there is an effect of game 1 on game 2. With increasing selection intensity, the fixation probability of a single individual playing $A_{1}^{1}A_{1}^{2}$ strategy on the edge $A_{1}^{1}A_{1}^{2}⇄A_{1}^{1}A_{2}^{2}$, i.e. $\rho_{A_{1}^{1}A_{1}^{2},\;A_{1}^{1}A_{2}^{2}}$ is expected to decrease (electronic supplementary material, figure A.5). However, this decrease is different for the two methods and for the fixation probability of an individual with strategy 1 playing only game *A*^2^, i.e. $\rho_{A_{1}^{2},\;A_{2}^{2}}$. Method I gives a higher value of $\rho_{A_{1}^{1}A_{1}^{2},\;A_{1}^{1}A_{2}^{2}}$ as compared to $\rho_{A_{1}^{2},\;A_{2}^{2}}$, whereas Method II shows that $\rho_{A_{1}^{1}A_{1}^{2},\;A_{1}^{1}A_{2}^{2}}$ is lower than $\rho_{A_{1}^{2},\;A_{2}^{2}}$ with increasing selection intensity. This means that while in general the fixation probabilities for the categorical type $A_{1}^{1}\;A_{1}^{2}$ decrease, it is even harder for $A_{1}^{1}\;A_{1}^{2}$ to reach fixation in the scenario where all the payoffs are first added and then converted to fitness as opposed to if the payoffs are first mapped and then added together. The difference can be explained by the difference in the baseline fitness between the two methods. The baseline fitness is provided by the game which the edge is independent of, in the case of electronic supplementary material, figure A.5, game *A*^1^. In the electronic supplementary material, we calculate the difference between the two methods and show how this difference changes according to the different baseline fitness. For a large number of games, the difference is independent of the number of games.

Fixation probability is a crucial property of stochastic evolutionary game dynamics. Instead of merely looking at the fixation probabilities of certain types or strategies in a game, we have expanded the method for analysing the ‘categorical types’ in the MGD. Therefore, even for multiple games in finite populations, it might be possible to derive the long-term average dynamics [28,36] of entities playing a combination of different roles (strategies) in various interactions (games).

### (c) Territorial defence versus group hunts

We can find numerous applications of the multiple games concept not only in economics and cultural evolution [37] but also in classical ecology and evolutionary biology. As an illustration of our methodology, we choose to focus on the lioness example described in the Introduction. An explanation involving multiple games was already hypothesized in [18]. We shall consider two games: the territorial defence and a hunting game. The first game is a public goods game (PGG) with loners (*Lo*, not participating in the defence), leaders (*Le*, cooperators), and laggards (*La*, defectors). The cooperators patrol the territory together and thus provide an enhanced benefit of better protection via numbers. The loners can protect the territory only by themselves and get limited benefit out of it (less than the cooperators). The defectors take part in patrolling but lag thus benefiting from the interaction without contributing. The payoffs for these strategies are

3.7 $$\pi_{Le}=\frac{r_{1}c_{1}k}{d_{1}}-c_{1};\;\pi_{La}=\frac{r_{1}c_{1}k}{d_{1}};\;\pi_{Lo}=c_{1}\sigma .$$

**Figure 5.**
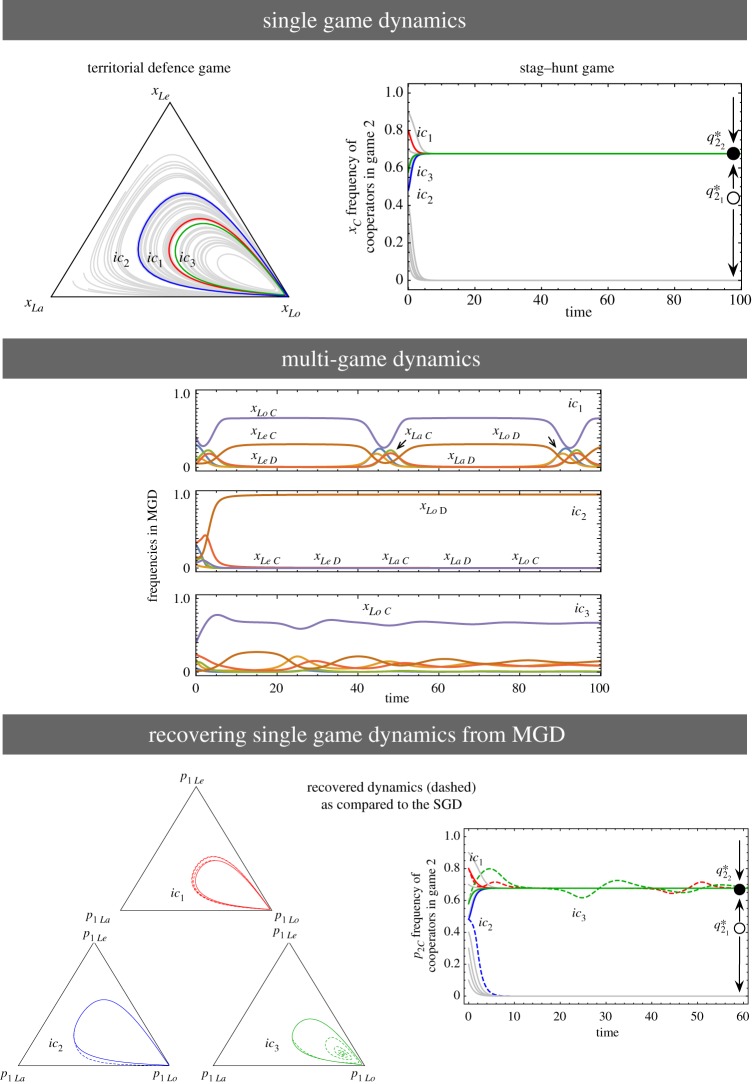
Lionesses in territory defence and stag–hunt games. The SGD of the games are plotted in the top panel. The leader *Le*, laggard *La*, and loner *Lo* are the strategies in the territorial defence game. Cooperation *C* and defection *D* are the strategies for the stag hunt. The grey lines are trajectories from random initial conditions to observe the SGDs. We choose three trajectories having initial conditions *ic*_1_, *ic*_2_, and *ic*_3_ to track the SGDs and MGD. Homoclinic orbits that emerge from and lead to *x*_*Lo*_ can be seen in the SGD of game 1. The SGD of game 2 shows an unstable equilibrium at ($q_{2_{1}}^{*}$) and a stable one at $q_{2_{2}}^{*}$. The MGD consists of six categorical types *x*_*LeC*_, *x*_*LaC*_, *x*_*LoC*_, *x*_*LeD*_, *x*_*LaD*_, and *x*_*LoD*_. In the middle panel, the time evolution of the categorical types is plotted. For *ic*_1_, we recover oscillatory dynamics but different dynamics as well as equilibria emerge for other initial conditions. In the last row, we show the recovered SGDs (plotted in dashed lines) from the MGD in comparison with the original SGDs (plotted with solid lines). For the recovered territorial defence game, the initial conditions *ic*_2_ and *ic*_3_ do not end up in the homoclinic cycle as in the SGDs; the equilibrium solution and dynamics in the multi-game is different from the SGD. For *ic*_1_ and *ic*_3_, cooperation in game 2, i.e. *p*_2 *C*_ does not reach a static equilibrium but oscillates. On the other hand, *ic*_2_ goes extinct; a complete switch of equilibrium as compared to the SGD. So the addition of games changes the dynamics as well as stability of both the games for certain initial conditions. (Online version in colour.)

For territory defence, we set the number of individuals patrolling *d*_1_, with the cost of cooperation *c*_1_. The parameter *k* is the number of leaders (or cooperators). Here, *r*_1_ (1 < *r*_1_ < *d*_1_) is the common pool’s interest rate or an enhancement factor and *σ* (0 < *σ* < *r*_1_ − 1) is the small and fixed payoff of loners. The SGD for *d*_1_ = 12, *r*_1_ = 3, *c*_1_ = 1, and *σ* = 1 is shown in figure 5 as in [38]. The homoclinic cycles show the coexistence of all the types: leaders, laggards, and loners as discussed in the Introduction.

The second game is a hunting game (stag–hunt game) with cooperators and defectors. In cooperative hunting among lionesses, the ‘wings’ attack a prey and force them to move forward. The prey ends up running towards the lionesses called ‘centres’ lurking to catch it [39]. Clearly, two players are not enough for these games. For the two strategies of this multiplayer stag–hunt game, the payoffs are calculated as per [9]

3.8 $$\pi_{C}=\frac{r_{2}c_{2}j}{d_{2}}\theta (j-M)-c_{2}\;\text{and}\;\pi_{D}=\frac{r_{2}c_{2}j}{d_{2}}\theta (j-M),$$

where *θ*(*z*) is the Heaviside step function, i.e. *θ*(*z* < 0) = 0 and *θ*(*z* ≥ 0) = 1. The number of cooperators *j* each pay a cost *c*_2_. The enhancement factor for game 2 is given by *r*_2_. The value *M* is the minimum threshold number of players required to produce public good. The SGD for this scenario is depicted in figure 5. For specific parameter values, *d*_2_ = 20, *M* = 10, *c*_2_ = 1, and *r*_2_ = 12, we observe two internal equilibrium solutions of the replicator dynamics [9].

Combining the stag hunt with the territorial defence game, the recovered dynamics from the MGD does not necessarily reflect the SGDs. Certain trajectories can become non-oscillatory resulting in the dominance of one of the strategies (*ic*_2_) or the coexistence of all but in a static equilibrium (*ic*_3_). For the stag–hunt game, we even see a complete switch of equilibrium (*ic*_2_), as in figure 4. The combination of the two games can change not just the dynamics but also the equilibria of both the games for certain initial conditions (figure 5).

From the MGD shown in figure 5, we see that judging a lioness by her action in one game does not complete the picture. An apparent cheater lioness in one game, can be a cooperator in another. For *ic*_2_, *x*_*LoD*_ reaches fixation but for *ic*_1_ the timing of observation matters. A lioness’ entire story can only be told by looking at her ‘categorical type’ which informs us about the combined effect of playing all games as postulated by empirical observations [18]. Adding other games like cooperative breeding, nursing, or mating may also provide a better comprehension.

## 4. Conclusion

Nature is composed of many interactions in different contexts (games) [40]. The games consist of different players and strategy sets. In its lifetime, an individual plays many parts (in various games). We have devised a method to combine the various multiplayer multi-strategy games that individuals play with an aim of developing realistic evolutionary game theoretic models. For infinite populations, we provide a replicator equation which can encapsulate multiple games with multiple players and strategies. For finite populations, we show that the fixation probabilities depend on the details of the particular model at hand and especially how the payoffs are converted to fitness.

Just as biological and social analogies of multiplayer evolutionary games can be found aplenty, the case for considering multiple multiplayer games is strong. We have discussed an application of our theory using the territorial defence and hunting behaviour of lionesses. The example highlights the fact that behaviour needs to be analysed in the light of complex multiple interaction contexts. On a smaller scale, the gut microbiota is a complex system which is capable of showing a variety of stable states, often a dynamic stability [14,41]. The different microbes within the gut community definitely interact in a variety of ways within themselves but each also interacts with the host in a unique manner. Within species and between species interactions, together, have the potential to dictate the evolutionary course of all involved species [42]. These interactions can certainly be interpreted as multiple games, each with a number of strategies and (immensely) multiplayer games. On the population genetics level, as an extension to previous work [43], multiple games and multi-strategies can be seen as multiple loci with several alleles. The case for two loci (or games) having two strategies [31], and 3-strategy games [8] has been previously investigated. Now with our inclusion of multiplayer games, we can also investigate polyploidy [44]. Considering recombination at this point would be crucial since it has been shown that under recombination the dynamics of multiple games would converge to the Wright manifold and thus to the SGD as in [45]. Deciphering the linkage between strategies used across multiple games could then be an exciting avenue for future research.

In finite populations, we have developed two methods to map the payoffs to the fitnesses. These two methods produce different fixation probability values for a particular selection intensity (electronic supplementary material, figure A.5). Both methods can have different biological justification. For example, all the actions leading to a brood produced during a season could be the culmination of all payoffs converted to fitness and then added to give the lifetime fitness—this is akin to Method I. On the other hand, in Method II, the payoffs through all breeding seasons would be summed up and then mapped to the lifetime fitness. The methods produce different results as compared with just one game (or even when the game is combined with another neutral game). Thus, even under finite populations, MGDs are different from SGDs and our formulation can be used to study multiple games in finite populations.

In a nutshell, our analysis reveals that the outcomes from multiplayer 2-strategy games are similar to previous results [31], where the MGD can be characterized by the separate analysis of the individual games. However, when the games have at least three pure strategies, different dynamics emerge [8]. This dynamical (in)consistency has already been pointed out [31,32] as ‘serious since it goes to the heart of the evolutionary approach’ [32]. With the diverse use of multiplayer games in social evolution (e.g. tragedy of the commons) and in biology, the problem is only exacerbated due to the potential existence of multiple internal steady states. For such cases, a fully comprehensive study of the initial conditions is a potential future project (as in figures 4 and 5). Even though complicated dynamics can still be captured by the relatively simple replicator-like equations and fixation probabilities, vast domains in the multiple games space remain unexplored.

## Supplementary Material

Electronic Supplementary Material: Evolutionary dynamics of multiple games

## Data accessibility

This article has no additional data.
