# Supplementary material for: Evolutionary dynamics of complex multiple games
Source: Proc Biol Sci. 2019 Jun 26;286(1905):20190900. doi: 10.1098/rspb.2019.0900 (PMC6599991; doi:10.1098/rspb.2019.0900)
Supplement: Electronic Supplementary Material: Evolutionary dynamics of multiple games [file rspb20190900supp1.pdf]

# Electronic Supplementary Material: Evolutionary dynamics of complex multiple games

Vandana Revathi Venkateswaran and Chaitanya S. Gokhale

Research Group for Theoretical Models of Eco-evolutionary Dynamics
Max Planck Institute for Evolutionary Biology
Department of Evolutionary Theory
August Thienemann Str. 2, 24306, Plön, Germany
Email: gokhale@evolbio.mpg.de

# 1 Infinite population

## 1.1 Single Game Dynamics (SGD)

### A two player replicator approach

Consider a $2 \times 2$ (two player two strategy) payoff matrix (A.1) : There are two players and each of them can adopt two strategies. The two types of strategies they could employ are 1 and 2 and their respective frequencies are $x_1$ and $x_2$.

$$\begin{array}{c} \\ 1 \\ 2 \end{array} \begin{array}{c} \begin{array}{cc} 1 & \quad 2 \end{array} \\ \begin{pmatrix} a_{1,(1,0)} & a_{1,(0,1)} \\ a_{2,(1,0)} & a_{2,(0,1)} \end{pmatrix} \end{array} \tag{A.1}$$

In matrix A.1, we write the elements in the form $a_{i,\alpha}$, where $i$ is the strategy of the focal player. Using multiindex notation, $\alpha$, is a vector written as $\alpha = (\alpha_1, \alpha_2)$, together representing the group composition. The average payoffs of the two strategies are given by $f_1 = a_{1,(1,0)}x_1 + a_{1,(0,1)}x_2$ and $f_2 = a_{2,(1,0)}x_1 + a_{2,(0,1)}x_2$. The replicator equation Eq. (A.2) [1, 2] describes the change in frequency $x_i$ of strategy $i$ over time.

$$\dot{x}_i = x_i[(f_i - \phi)] \tag{A.2}$$

where $f_i$ is the fitness of strategy $i$ and $\phi$ is the average fitness. For an infinitely large population size we have $x_1 = x$, $x_2 = 1 - x$ Thus the replicator equation for the change in the

frequency of strategy 1 is,

$$\begin{aligned}\dot{x} &= x(1-x)(f_1 - f_2)\\ &= x(1-x)[(a_{1,(1,0)} - a_{1,(0,1)} - a_{2,(1,0)} + a_{2,(0,1)})x + a_{2,(1,0)} - a_{2,(0,1)}].\end{aligned} \tag{A.3}$$

Apart from the trivial fixed points ($x = 0$ and $x = 1$), there is an internal equilibrium given by,

$$\mathbf{x}^\star = \frac{a_{2,(0,1)} - a_{2,(1,0)}}{a_{1,(1,0)} - a_{1,(0,1)} - a_{2,(1,0)} + a_{2,(0,1)}}. \tag{A.4}$$

### Multiplayer games

We now extend the dynamics to multiplayer games [3]. The payoff matrix (A.5), represents a three player ($d = 3$) two strategy ($n = 2$) game; a $2 \times 2 \times 2$ game.

$$\begin{array}{c} \\ 1 \\ 2 \end{array} \begin{array}{c} \begin{array}{ccc} 11 & 12 & 22 \end{array} \\ \begin{pmatrix} a_{1,(2,0)} & a_{1,(1,1)} & a_{1,(0,2)} \\ a_{2,(2,0)} & a_{2,(1,1)} & a_{2,(0,2)} \end{pmatrix} \end{array} \tag{A.5}$$

The rows correspond to the focal player. Focal player interacting with two other players, both with strategy 1 will receive a payoff $a_{1,(2,0)}$. While interacting with a one strategy 1 player and a strategy 2 player, he will get $a_{1,(1,1)}$. When interacting with two other strategy 2 individuals, the payoff is equal to $a_{1,(0,2)}$. Assuming that the order of players does not matter, the average payoffs (or in this case, the fitnesses) will be,

$$\begin{aligned} f_1 &= x^2 a_{1,(2,0)} + 2x(1-x)a_{1,(1,1)} + (1-x)^2 a_{1,(0,2)} \\ f_2 &= x^2 a_{2,(2,0)} + 2x(1-x)a_{2,(1,1)} + (1-x)^2 a_{2,(0,2)}. \end{aligned} \tag{A.6}$$

The replicator equation in this case is given by,

$$\begin{aligned} \dot{x} = x(1-x)((a_{1,(0,2)} - 2a_{1,(1,1)} + a_{1,(2,0)} - a_{2,(0,2)} + 2a_{2,(1,1)} - a_{2,(2,0)})x^2 \\ +(-a_{1,(0,2)} + a_{1,(1,1)} + a_{2,(0,2)} - a_{2,(1,1)})2x + a_{1,(0,2)} - a_{2,(0,2)}). \end{aligned} \tag{A.7}$$

The quadratic $x^2$ term in Eq. (A.7) can give rise to a maximum of two interior fixed points. In general, for a $d$-player two strategy game, the replicator equation can result in $d-1$ interior fixed points (maximum). For an $n$ strategy $d$-player game, the maximum number of internal equilibria is $(d-1)^{(n-1)}$ as shown in [4].

## 1.2 Multi Game Dynamics (MGD)

### Linear combination of two $2 \times 2$ games

To start looking into the dynamics of combinations of games i.e. Multi Game Dynamics (MGD) in contrast with the Single Game Dynamics (SGD), consider the example: two games

with two strategies in each. Let the payoff matrix of Game 1 and Game 2 be,

$$
\begin{array}{cc}
 & \begin{array}{cc} A_1^1 & A_2^1 \end{array} \\
A^1 = \begin{array}{c} A_1^1 \\ A_2^1 \end{array} & \begin{pmatrix} a^1_{1,(1,0)} & a^1_{1,(0,1)} \\ a^1_{2,(1,0)} & a^1_{2,(0,1)} \end{pmatrix}
\end{array}
\qquad
\begin{array}{cc}
 & \begin{array}{cc} A_1^2 & A_2^2 \end{array} \\
A^2 = \begin{array}{c} A_1^2 \\ A_2^2 \end{array} & \begin{pmatrix} a^2_{1,(1,0)} & a^2_{1,(0,1)} \\ a^2_{2,(1,0)} & a^2_{2,(0,1)} \end{pmatrix}
\end{array}
$$

The individuals can be partitioned into four classes. Individuals playing strategy $1$ in game $A^1$ and game $A^2$, strategy $1$ in $A^1$ and $2$ in $A^2$, strategy $2$ in $A^1$ and $1$ in $A^2$, and strategy $2$ in $A^1$ and $A^2$. So, there are four types of strategies, $A_1^1A_1^2$, $A_1^1A_2^2$, $A_2^1A_1^2$ and $A_2^1A_2^2$. We refer to them as "categorical types". Their respective frequencies are written as $x_{11}$, $x_{12}$, $x_{21}$ and $x_{22}$. We shall now use a new notation, $p_{ji_j}$ or playing strategy $i_j$ in game $j$, which is just a variable transformation that can be written as (here, $i_j \in \{1, 2\}$ and $j \in \{1, 2\}$),

$$
\begin{aligned}
p_{11} &= x_{11} + x_{12} \\
p_{12} &= x_{21} + x_{22} \\
p_{21} &= x_{11} + x_{21} \\
p_{22} &= x_{12} + x_{22}.
\end{aligned}
\tag{A.8}
$$

The fitnesses for playing strategy $i_j$ in game $j$ can be written out as,

$$
\begin{aligned}
f_{11} &= x_{11}\ a^1_{1,(1,0)} + x_{12}\ a^1_{1,(1,0)} + x_{21}\ a^1_{1,(0,1)} + x_{22}\ a^1_{1,(0,1)} \\
f_{12} &= x_{11}\ a^1_{2,(1,0)} + x_{12}\ a^1_{2,(1,0)} + x_{21}\ a^1_{2,(0,1)} + x_{22}\ a^1_{2,(0,1)} \\
f_{21} &= x_{11}\ a^2_{1,(1,0)} + x_{12}\ a^2_{1,(0,1)} + x_{21}\ a^2_{1,(1,0)} + x_{22}\ a^2_{1,(0,1)} \\
f_{22} &= x_{11}\ a^2_{2,(1,0)} + x_{12}\ a^2_{2,(0,1)} + x_{21}\ a^2_{2,(1,0)} + x_{22}\ a^2_{2,(0,1)}.
\end{aligned}
\tag{A.9}
$$

A crucial assumption here is that the effective average payoff is a linear composite of the constituent games. The replicator dynamics will be given by the following set of coupled different differential equations:

$$
\begin{aligned}
\dot{x_{11}} &= x_{11}[(f_{11} + f_{21}) - \phi] \\
\dot{x_{12}} &= x_{12}[(f_{11} + f_{22}) - \phi] \\
\dot{x_{21}} &= x_{21}[(f_{12} + f_{21}) - \phi] \\
\dot{x_{22}} &= x_{22}[(f_{12} + f_{22}) - \phi].
\end{aligned}
\tag{A.10}
$$

The average fitness $\phi$ is given by,

$$
\begin{aligned}
\phi &= x_{11}(f_{11} + f_{21}) + x_{12}(f_{11} + f_{22}) + x_{21}(f_{12} + f_{21}) + x_{22}(f_{12} + f_{22}) \\
&= f_{11}(x_{11} + x_{12}) + f_{12}(x_{21} + x_{22}) + f_{21}(x_{11} + x_{21}) + f_{22}(x_{12} + x_{22}) \\
&= f_{11}\ p_{11} + f_{12}\ p_{12} + f_{21}\ p_{21} + f_{22}\ p_{22}.
\end{aligned}
\tag{A.11}
$$

The single games' dynamics and their multi game dynamics will be the same or in other words, an MGD can be separated back into all its SGDs if $p_{ji_j} = x_{i_j}\ \forall\ i_j$ in a game $j$, for all $N$ games. At times, even if this equality holds, the trajectories in the MGD space might be different from the SGD space. Both these cases are shown in the examples in the main article. A previous study with two player games with two strategies [5], showed that the SGDs can be separated from their MGD. The dynamics lie on the generalized invariant *manifold*. [1, 6] in the $S_4$ simplex which is given by $W_K = \{x \in S_4 \mid x_{11}x_{22} = Kx_{12}x_{21}\}$ for $K > 0$. When $K = 1$, we have $W = \{x \in S_4 \mid x_{11}x_{22} = x_{12}x_{21}\}$ which is the *Wright manifold*. The Wright manifold $W_K$ [6, 1] is a population dynamic concept. The states belonging to the Wright manifold are for the population in linkage equilibrium i.e. the games (or loci/traits, in biology) are inherited completely independently in each generation. Thus, on this manifold, MGD can be separated back into the SGDs of the constituent games. The attractor for a combination of two 2-player games having two strategies each is a line $E$, an evolutionarily stable set [5]. The point where the line $E$ intersects the Wright manifold indicates a rest point. All the trajectories in the simplex depicting the MGD fall onto an attractor given by a line (ES set) on $W_K$. The dynamics on $W_K$ and the trajectories on each $W_K$ were analyzed in the same study [5] using methods used in dynamical systems to show they are qualitatively the same as on the Wright manifold.

However, for multiple games having more than two strategies in at least one game, the MGD cannot be separated even into a linear combination of the constituent SGDs unless they are on $W$ [7]. Increase in the number of games and the number of strategies increases the dimension of MGD simplex. This high dimensional space of MGD, which would be equal to $\Sigma_{i=1}^{N}(m_j - 1)$ (where $N$ is the number of games and $m_j$ is the number of strategies in a game $j$), is densely packed with manifolds. All the manifolds are non-intersecting while $W$ is the invariant. Even for a simplified example of 2 games each with $m_1$ and $m_2$ number of strategies the generalised invariant manifold is given by $W_K = \{x \in \Delta^{m_1 \times m_2} | x_{i,k}x_{j,l} = K_{ik,jl}\ x_{i,l}x_{j,k}\ \forall\ 1 \leq i, j \leq m_1, 1 \leq k, l \leq m_2\}$ where $K = \{K_{ik,jl}\}$ is a set of positive constants for which $W_K$ is a non-empty set. When $K_{ik,jl} = 1$, we have the Wright manifold on which the MGD can be separated back into its SGDs. While combining two 2-player games with three strategies [7], the evolutionarily stable set $E$ would be in a four-dimensional hyperplane [6]. So while combining many games, even if one individual game has more than two strategies, the ES set may no longer be a line. It would be a hyperplane in the $W_K$ hyperspace. Thus, it is important to know on which manifold the initial conditions are, for only if they start from the Wright manifold $W$, will the dynamics be a perfect match to the SGDs [7].

If the initial condition is not on $W$, if the strategies between the different games are allowed

to recombine then the dynamics converges to $W$. While the relationship between strategies under recombination is genetically plausible, for phenotypic strategies, social learning or horizontal adoption of traits could have a similar effect [8, 9].

# 2 Finite population

## 2.1 Single game dynamics

In a population of size $Z$ consisting of strategy 1 and strategy 2 players, the probability that one of the strategies, say 1, fixates, is given by the fixation probability $\rho_1$. An individual is chosen proportional to its fitness to reproduce an identical offspring. Another individual is chosen randomly and discarded from the group. Therefore, the group size is kept at a constant value $Z$. Fitness of a strategy $s$ can be a linear function of its average payoff $\pi_s$ i.e $f_s = 1 - w + w\pi_s$. In a population that has $i$ strategy 1 players, the fitnesses can be used to calculate the transition probabilities $T_i^+$ and $T_i^-$ for the number of type 1 players to increase and decrease by one, respectively.

$$\begin{aligned} T_i^+ &= \frac{if_1}{if_1 + (Z-i)f_2}\frac{Z-i}{Z} \\ T_i^- &= \frac{(Z-i)f_2}{if_1 + (Z-i)f_2}\frac{i}{Z}. \end{aligned} \tag{A.12}$$

With probability $1 - T_i^+ - T_i^-$ the system does not change. Using the transition probabilities, the fixation probability can be calculated [2, 10] to be,

$$\rho_1 = \frac{1}{1 + \sum_{m=1}^{Z-1} \prod_{i=1}^{m} \frac{T_i^-}{T_i^+}}. \tag{A.13}$$

Since $\frac{T_i^-}{T_i^+} = \frac{f_2}{f_1} = \frac{1-w+w\pi_2}{1-w+w\pi_1} \approx 1 - w(\pi_1 - \pi_2)$ for selection intensity $w \ll 1$ i.e. weak selection. Therefore,

$$\rho_1 \approx \frac{1}{1 + \sum_{m=1}^{Z-1} \prod_{i=1}^{m} 1 - w(\pi_1 - \pi_2)}. \tag{A.14}$$

For a $d$-player game, the payoffs are obtained using a hypergeometric distribution given by,

$$H(k, d; i, Z) = \frac{\binom{i-1}{k}\binom{Z-i}{d-1-k}}{\binom{Z-1}{d-1}}. \tag{A.15}$$

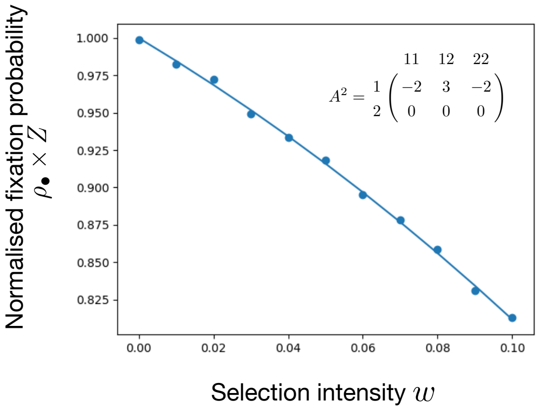


Figure A.1: **Fixation probability for a single individual playing strategy** $1$ **varying with selection intensity for a three player game having two strategies.** For the game shown in this figure, the payoff of strategy 2 is greater than strategy 1 ($\pi_2 > \pi_1$), the fixation probability decreases, according to equation (A.17). The results from analytics and simulations (averaged over $10^6$ realizations) are plotted as solid lines and solid circles, respectively.

Thus,

$$\begin{aligned} \pi_1 &= \sum_{k=0}^{d-1} \frac{\binom{i-1}{k}\binom{Z-i}{d-1-k}}{\binom{Z-1}{d-1}} a_{1,\alpha} \\ \pi_2 &= \sum_{k=0}^{d-1} \frac{\binom{i}{k}\binom{Z-i-1}{d-1-k}}{\binom{Z-1}{d-1}} a_{2,\alpha}. \end{aligned} \tag{A.16}$$

Maintaining weak selection, then from [4] we have,

$$\rho_1 \approx \frac{1}{Z} + \frac{w}{Z^2} \sum_{m=1}^{Z-1} \sum_{i=1}^{m} (\pi_1 - \pi_2). \tag{A.17}$$

Figure A.1 contains the fixation probabilities of strategy 1 with respect to varying selection intensities for a three player game with two strategies.

## 2.2 Multiple game dynamics

We begin with the same example that was used to explain the combination of two $d$-player games where both games have two strategies; and use the same notations for a finite population of size $Z$. The population consists of individuals of four types : $A_1^1 A_1^2$, $A_1^1 A_2^2$, $A_2^1 A_1^2$ and $A_2^1 A_2^2$. The combined dynamics results in an $S_4$ simplex as shown in Fig. A.2. We perform pairwise comparisons for all the edges of the simplex. On a particular edge, only the two

$$A^1 = \begin{array}{c} \\ 1 \\ 2 \end{array} \begin{array}{c} \begin{array}{cc} 1 & 2 \end{array} \\ \begin{pmatrix} -1 & 1 \\ 0 & 0 \end{pmatrix} \end{array} \qquad A^2 = \begin{array}{c} \\ 1 \\ 2 \end{array} \begin{array}{c} \begin{array}{ccc} 11 & 12 & 22 \end{array} \\ \begin{pmatrix} -2 & 3 & -2 \\ 0 & 0 & 0 \end{pmatrix} \end{array}$$

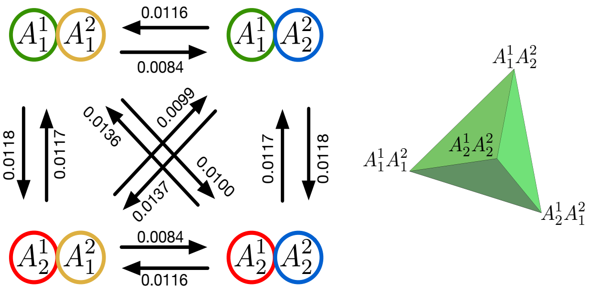


Figure A.2: **Fixation probabilities over pure strategies.** Figure shows the fixation probabilities and the direction of selection between the vertices in a tetrahedron (which contains the MGD of the two games $A^1$ and $A^2$ shown in the matrices). Here selection intensity $w = 0.01$ and population size $Z = 100$. It has been assumed that both the games have the same selection intensity and hence the average payoffs have been added first and then the mapping (linear or exponential mapping from payoffs to fitness) has been performed i.e. Method II (Method I would produce a different figure). For the edges where one of the games does not change (e.g. $A_1^1, A_1^2 \rightleftarrows A_1^1, A_2^2$), only one of the game (here game 2) matters and hence the fixation probabilities are the same as if *only* one game.

vertex strategies are present. Let us start with the edge containing $x_{11}$ and $x_{12}$ vertices. If there are $\gamma_{11}$ individuals playing strategy $A_1^1A_1^2$, then there are $\gamma_{12} = Z - \gamma_{11}$ individuals of type $A_1^1A_2^2$. The number of $A_2^1A_1^2$ and $A_2^1A_2^2$ individuals i.e. $\gamma_{21}$ and $\gamma_{22}$ is zero. In the individual games, the number of players adopting strategy $i_j$ in a game $j$ is given by $p_{ji_j}$. Since we are looking at the edge with $A_1^1A_1^2$ and $A_1^1A_2^2$ individuals, we have

$$\begin{aligned} p_{11} &= \gamma_{11} + \gamma_{12} = Z \\ p_{12} &= \gamma_{21} + \gamma_{22} = 0 \\ p_{21} &= \gamma_{11} + \gamma_{21} = \gamma_{11} \\ p_{22} &= \gamma_{12} + \gamma_{22} = Z - \gamma_{11}. \end{aligned} \tag{A.18}$$

In contrast to the binomial distribution which is used for infinite populations where the draws can be considered independent, the hypergeometric distribution was used for sampling without replacement in the case of finite populations [4, 11]. For infinite population, we used the multinomial distribution to calculate the average payoffs for a combination of $N$ multiplayer games in an infinite population size. Therefore, for finite populations, we shall use the multivariate hypergeometric distribution. For a population of size $Z$ containing $\gamma_{11}$ type $A_1^1A_1^2$ and $Z - \gamma_{11}$ type $A_1^1A_2^2$ individuals, the average payoffs $\pi_{ji_j}$ for playing strategy $i_j$ in game $j$ (in our example, $i_j \in \{1, 2\}$ and $j \in \{1, 2\}$) are

$$\begin{aligned} \pi_{11} &= \sum_{|k|=d_1-1} \frac{\binom{p_{11}-1}{k_1}\binom{p_{12}}{k_2}}{\binom{Z-1}{d_1-1}} a^1_{1,k} \\ \pi_{12} &= \sum_{|k|=d_1-1} \frac{\binom{p_{11}}{k_1}\binom{p_{12}-1}{k_2}}{\binom{Z-1}{d_1-1}} a^1_{2,k} \\ \pi_{21} &= \sum_{|k|=d_2-1} \frac{\binom{p_{21}-1}{k_1}\binom{p_{22}}{k_2}}{\binom{Z-1}{d_2-1}} a^2_{1,k} \\ \pi_{22} &= \sum_{|k|=d_2-1} \frac{\binom{p_{21}}{k_1}\binom{p_{22}-1}{k_2}}{\binom{Z-1}{d_2-1}} a^2_{2,k}. \end{aligned} \tag{A.19}$$

In general, for $N$ multi-strategy $d$-player games,

$$\pi_{ji_j} = \sum_{|k|=d_j-1} \frac{\binom{p_{ji_j}-1}{k_{i_j}} \prod_{n=1,n\neq i_j}^{m_j} \binom{p_{jn}}{k_n}}{\binom{Z-1}{d_j-1}} a^j_{i_j,k}. \tag{A.20}$$

We can calculate the fitnesses using linear or exponential mapping. If $w_j$ is the intensity

of selection in game $j$, then

$$f_{ji_j} = \begin{cases} 1 - w_j + w_j \pi_{ji_j} & \text{for linear mapping} \\ e^{w_j \pi_{ji_j}} & \text{for exponential mapping.} \end{cases} \tag{A.21}$$

Thus, in the combined dynamics, the fitness (assuming it to be additive) of type $A^1_{i_1} A^2_{i_2} ... A^N_{i_N}$ is

$$F_{i_1 i_2 .... i_N} = \sum_{j=1}^{N} f_{ji_j}. \tag{A.22}$$

If we are looking at an edge with types $A^1_{i_1} A^2_{i_2} ... A^N_{i_N}$ and $A^1_{h_1} A^2_{h_2} ... A^N_{h_N}$, the transition probability $T^+_\gamma$ for type $A^1_{i_1} A^2_{i_2} ... A^N_{i_N}$ to increase from $\gamma$ to $\gamma + 1$ (and type $A^1_{h_1} A^2_{h_2} ... A^N_{h_N}$ to be randomly selected for death) is

$$T^+_\gamma = \frac{\gamma F_{i_1 i_2 .... i_N}}{\gamma F_{i_1 i_2 3 .... i_N} + (Z - \gamma) F_{h_1 h_2 .... h_N}} \frac{Z - \gamma}{Z}. \tag{A.23}$$

Likewise, $T^-_\gamma$ will be

$$T^-_\gamma = \frac{(Z - \gamma) F_{h_1 h_2 .... h_N}}{\gamma F_{i_1 i_2 .... i_N} + (Z - \gamma) F_{h_1 h_2 .... h_N}} \frac{\gamma}{Z}. \tag{A.24}$$

So, for a $A^1_{i_1} A^2_{i_2} ... A^N_{i_N}$ and $A^1_{h_1} A^2_{h_2} ... A^N_{h_N}$ edge, the fixation probability $\rho_{A^1_{i_1} A^2_{i_2} ... A^N_{i_N}}$ of type $A^1_{i_1} A^2_{i_2} ... A^N_{i_N}$ is

$$\rho_{A^1_{i_1} A^2_{i_2} ... A^N_{i_N},\, A^1_{h_1} A^2_{h_2} ... A^N_{h_N}} = \frac{1}{1 + \sum_{m=1}^{Z-1} \prod_{\gamma=1}^{m} \frac{T^-_\gamma}{T^+_\gamma}}. \tag{A.25}$$

**Method I**

As $\frac{T^-_\gamma}{T^+_\gamma} = \frac{F_{h_1 h_2 h_3 .... h_N}}{F_{i_1 i_2 i_3 .... i_N}}$, Eq. (A.25) can be written as,

$$\begin{aligned} \rho_{A^1_{i_1} A^2_{i_2} ... A^N_{i_N},\, A^1_{h_1} A^2_{h_2} ... A^N_{h_N}} &= \frac{1}{1 + \sum_{m=1}^{Z-1} \prod_{\gamma=1}^{m} \frac{F_{h_1 h_2 h_3 .... h_N}}{F_{i_1 i_2 i_3 .... i_N}}} \\ &= \frac{1}{1 + \sum_{m=1}^{Z-1} \prod_{\gamma=1}^{m} \frac{\sum_{j=1}^{N} f_{jh_j}}{\sum_{j=1}^{N} f_{ji_j}}} \\ &= \frac{1}{1 + \sum_{m=1}^{Z-1} \prod_{\gamma=1}^{m} \left( \frac{N + \sum_{j=1}^{N} -w_j + w_j \pi_{jh_j}}{N + \sum_{j=1}^{N} -w_j + w_j \pi_{ji_j}} \right)}. \end{aligned} \tag{A.26}$$

where the fitness is obtained using a linear mapping. In order to further simplify the model, we consider that all games have the same selection intensity. In this case,

$$\begin{aligned}\rho_{A^1_{i_1}A^2_{i_2}\ldots A^N_{i_N},\,A^1_{h_1}A^2_{h_2}\ldots A^N_{h_N}} &= \frac{1}{1+\sum_{m=1}^{Z-1}\prod_{\gamma=1}^{m}\left(\frac{N-Nw+w(\sum_{j=1}^{N}\pi_{jh_j})}{N-Nw+w(\sum_{j=1}^{N}\pi_{ji_j})}\right)} \\ &= \frac{1}{1+\sum_{m=1}^{Z-1}\prod_{\gamma=1}^{m}\left(\frac{1-w+\frac{w}{N}(\sum_{j=1}^{N}\pi_{jh_j})}{1-w+\frac{w}{N}(\sum_{j=1}^{N}\pi_{ji_j})}\right)}.\end{aligned} \tag{A.27}$$

It is worth mentioning here that the assumption of having equal intensities for all games is strong. Many times, the selection on one game may be more intense than others. These have to be taken into account as it strengthens the precision of the model and Eq. (A.26) must be used in these scenarios. However for the sake of our analysis, we shall assume $w_j = w$ for all $j \in [0, N]$.

For weak selection intensity,

$$\begin{aligned}\rho_{A^1_{i_1}A^2_{i_2}\ldots A^N_{i_N},\,A^1_{h_1}A^2_{h_2}\ldots A^N_{h_N}} &\approx \frac{1}{1+\sum_{m=1}^{Z-1}\prod_{\gamma=1}^{m}[1-w\{1-\frac{(\sum_{j=1}^{N}\pi_{jh_j})}{N}\}]\times[1+w\{1-\frac{(\sum_{j=1}^{N}\pi_{ji_j})}{N}\}]} \\ &\approx \frac{1}{1+\sum_{m=1}^{Z-1}\prod_{\gamma=1}^{m}[1-\frac{w}{N}(\sum_{j=1}^{N}(\pi_{ji_j}-\pi_{jh_j}))]}.\end{aligned} \tag{A.28}$$

Eq. (A.28) can be written as,

$$\rho_{A^1_{i_1}A^2_{i_2}\ldots A^N_{i_N},\,A^1_{h_1}A^2_{h_2}\ldots A^N_{h_N}} \approx \frac{1}{Z-\frac{w}{N}\sum_{m=1}^{Z-1}\sum_{\gamma=1}^{m}(\sum_{j=1}^{N}(\pi_{ji_j}-\pi_{jh_j}))]}\,. \tag{A.29}$$

Following Taylor expansion and since $w \ll 1$, we get

$$\rho_{A^1_{i_1}A^2_{i_2}\ldots A^N_{i_N},\,A^1_{h_1}A^2_{h_2}\ldots A^N_{h_N}} \approx \underbrace{\frac{1}{Z}}_{\text{Under neutrality (w=0)}} + \frac{w}{NZ^2}[\sum_{m=1}^{Z-1}\sum_{\gamma=1}^{m}(\sum_{j=1}^{N}(\pi_{ji_j}-\pi_{jh_j}))]. \tag{A.30}$$

For $w = 0$ and $N = 1$ i.e. neutrality condition while there is only one game, the above equation is also equal to the classic neutral fixation probability $\frac{1}{Z}$ for single games. For $N = 1$ in Eq. (A.30) , we can retrieve Eq. (A.17) for a single multiplayer game i.e.

$$\rho_{A^1_{i_1},\,A^1_{h_1}} \approx \underbrace{\frac{1}{Z}}_{\text{Under neutrality}} + \frac{w}{Z^2}\sum_{m=1}^{Z-1}\sum_{\gamma=1}^{m}(\pi_{1i_1}-\pi_{1h_1}). \tag{A.31}$$

For $N = 2$ Eq. (A.28) becomes,

$$\rho_{A^1_{i_1}A^2_{i_2},\, A^1_{h_1}A^2_{h_2}} \approx \frac{1}{1+\sum_{m=1}^{Z-1}\prod_{\gamma=1}^{m} 1-\frac{w}{2}[(\pi_{1i_1}+\pi_{2i_2})-(\pi_{1h_1}+\pi_{2h_2})]}. \tag{A.32}$$

While looking at an edge for which, say, game 1 in both vertices has the same strategy and thus, we need to only look at differences in one game i.e. only game 2 matters ($\pi_{1i_1} = \pi_{1h_1}$),

$$\begin{aligned}\rho_{A^1_{i_1}A^2_{i_2},\, A^1_{h_1}A^2_{h_2}} &\approx \frac{1}{1+\sum_{m=1}^{Z-1}\prod_{\gamma=1}^{m} 1-\frac{w}{2}[(\pi_{2i_2}-\pi_{2h_2})]} \\ &= \frac{1}{Z}+\frac{w}{2Z^2}\sum_{m=1}^{Z-1}\sum_{i=1}^{m}(\pi_{2i_2}-\pi_{2h_2})\end{aligned} \tag{A.33}$$

We can make pairwise comparisons between all categorical types (all the edges of the $S_4$ simplex in containing the MGD of the two games with two strategies). Using these comparative fixation probabilities we can determine the flow of the dynamics over pure strategies as shown Fig. A.2.

### Method II

If all games have the same intensity, we could also add the payoffs first and then perform the fitness mappings, then $F_{i_1i_2i_3....i_N} = 1-w+w\left(\sum_{j=1}^{N}\pi_{ji_j}\right)$ and $F_{h_1h_2h_3....h_N} = 1-w+w\left(\sum_{j=1}^{N}\pi_{jh_j}\right)$. Thus, the combined fitness (of a vertex) is not just a sum of the fitnesses of strategies used in the inherent games (in that vertex). The combined fitness is obtained by summing the average payoffs of playing the respective strategies in the games involved in a particular vertex and using that to calculate the fitness of that vertex. Only the payoffs of the games that have the same selection intensity can be added together and mapped to fitness through this method. An example of a situation where the combined effect of the payoffs for the strategies of the games on that vertex leads to the combined fitness, would be in models of mating and sexual selection. Numerous interactions (parenting, mating, brooding) or games during a mating season decides the reproductive success or fitness of an individual during that period. This combination of games is not trivial as bringing all the smaller games into one larger game but we cannot always deconstruct the multi-game back to all the inherent single games. The fixation probability, Eq. (A.25), in this case will be,

$$\rho_{A^1_{i_1}A^2_{i_2}...A^N_{i_N},\, A^1_{h_1}A^2_{h_2}...A^N_{h_N}} = \frac{1}{1+\sum_{m=1}^{Z-1}\prod_{\gamma=1}^{m}\left(\frac{1-w+w(\sum_{j=1}^{N}\pi_{jh_j})}{1-w+w(\sum_{j=1}^{N}\pi_{ji_j})}\right)}. \tag{A.34}$$

For weak selection intensities,

$$\rho_{A^1_{i_1}A^2_{i_2}...A^N_{i_N},\, A^1_{h_1}A^2_{h_2}...A^N_{h_N}} \approx \frac{1}{1+\sum_{m=1}^{Z-1}\prod_{\gamma=1}^{m}\Big(1-w[1-(\sum_{j=1}^{N}\pi_{jh_j})]+w[1-(\sum_{j=1}^{N}\pi_{ji_j})]\Big)}$$
$$= \frac{1}{1+\sum_{m=1}^{Z-1}\prod_{\gamma=1}^{m}\Big(1-w[(\sum_{j=1}^{N}\pi_{ji_j}-(\sum_{j=1}^{N}\pi_{jh_j})]\Big)}. \quad (A.35)$$

and this can be further written as,

$$\rho_{A^1_{i_1}A^2_{i_2}...A^N_{i_N},\, A^1_{h_1}A^2_{h_2}...A^N_{h_N}} \approx \underbrace{\frac{1}{Z}}_{\text{Under neutrality (w=0)}} + \frac{w}{Z^2}[\sum_{m=1}^{Z-1}\sum_{\gamma=1}^{m}(\sum_{j=1}^{N}(\pi_{ji_j}-\pi_{jh_j}))]. \quad (A.36)$$

If we consider two games, then Eq. (A.35) will be reduced to

$$\rho_{A^1_{i_1}A^2_{i_2},\, A^1_{h_1}A^2_{h_2}} \approx \frac{1}{1+\sum_{m=1}^{Z-1}\prod_{\gamma=1}^{m}(1-w[(\pi_{1i_1}+\pi_{2i_2})-(\pi_{1h_1}+\pi_{2h_2})])}. \quad (A.37)$$

Here, if we look at an edge for which, say, game 1 in both vertices has the same strategy ($\pi_{1i_1}=\pi_{1h_1}$), then looking at differences in game 2 is what matters. In this scenario,

$$\rho_{A^1_{i_1}A^2_{i_2},\, A^1_{h_1}A^2_{h_2}} \approx \frac{1}{1+\sum_{m=1}^{Z-1}\prod_{\gamma=1}^{m}(1-w(\pi_{2i_2}-\pi_{2h_2}))}. \quad (A.38)$$

This corresponds to equation Eq. (A.14) for a single game with two strategies $i_1$ and $h_1$. This can also be written as ,

$$\rho_{A^1_{i_1}A^2_{i_2},\, A^1_{h_1}A^2_{h_2}} \approx \frac{1}{Z} + \frac{w}{Z^2}\sum_{m=1}^{Z-1}\sum_{i=1}^{m}(\pi_{2i_2}-\pi_{2h_2}) \quad (A.39)$$

and this is similar to Eq. (A.17) for single game dynamics. We can make pairwise comparisons between all categorical types (all the edges of the $S_4$ simplex in containing the MGD of the two games with two strategies). Using these comparative fixation probabilities we can determine the flow of the dynamics over pure strategies as shown Fig. A.2.

## Difference between Method I and II

The difference between Method I and II is given by,

$$|\,(\frac{1}{Z}+\frac{w}{Z^2}[\sum_{m=1}^{Z-1}\sum_{\gamma=1}^{m}(\sum_{j=1}^{N}(\pi_{ji_j}-\pi_{jh_j}))]) - (\frac{1}{Z}+\frac{w}{NZ^2}[\sum_{m=1}^{Z-1}\sum_{\gamma=1}^{m}(\sum_{j=1}^{N}(\pi_{ji_j}-\pi_{jh_j}))])\,|$$
$$=|\,\frac{w}{Z^2}[\sum_{m=1}^{Z-1}\sum_{\gamma=1}^{m}(\sum_{j=1}^{N}(\pi_{ji_j}-\pi_{jh_j}))].[1-\frac{1}{N}]\,|\,. \quad (A.40)$$

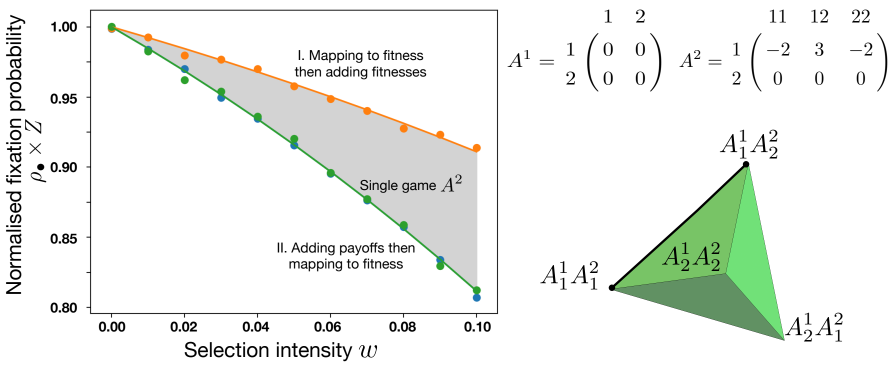


Figure A.3: **Fixation probability of a single individual playing $A_1^1A_1^2$ strategy on the edge $A_1^1A_1^2 \rightleftarrows A_1^1A_2^2$ varying with selection intensity for a combination of two games having two strategies each (special case $A^1$).** For a population of $Z = 10$ the fixation probabilities are normalised according to the neutral fixation probability, $\frac{1}{Z} = 0.1$. We look at the edge $A_1^1A_1^2 \rightleftarrows A_1^1A_2^2$ where $A^1$ is the same for both vertices i.e. neutral in both the vertices, and $A^2$ is what matters. The payoffs in Game $A^1$ are zero. Since the payoff of playing strategy 2 in $A^2$ is greater than playing strategy 1 ($\pi_{22} > \pi_{21}$), the fixation probability decreases as shown in the earlier sections of the ESM. The line labeled 'single game' corresponds to single game dynamics of $A^2$. The plots from Method I (mapping payoffs to fitnesses and then adding the fitnesses) and Method II (adding the payoffs first, and then mapping to fitness) for a combination of the two games $A^1$ and $A^2$. Since $\pi_{11}(= \pi_{12}) = 0$, results from Method II and the SGD of $A^2$ are the same. However, Method I shows a different result. Here, MGD differs from the SGD. Adding another game to $A^2$ modifies the dynamics. Thus, within the MGD, the two methods of mapping from payoffs to fitness i.e. Method I and Method II differ from each other (by Eq. A.41 shaded region). The difference is due to the different baseline payoffs that the different mappings produce. The results from analytics and stochastic simulations are plotted as solid lines and symbols, respectively. The simulations are averaged over $10^6$ realisations. Thus while looking at a combination of various games, there can be different methods of mapping and one needs to choose a mapping method that reflects their model best as they can bring about different results.

As $N$ increases, the difference between the two methods becomes independent of the number of games. For $N = 2$, if we look at an edge where game 1 at both vertices has the same strategy ($\pi_{1i_1} = \pi_{1h_1}$) then game 2 is what matters. Here, the difference between Methods I and II is the difference between the equations (A.39) and (A.33) which is equal to $\frac{w}{Z^2}[\sum_{m=1}^{Z-1}\sum_{\gamma=1}^{m}(\pi_{2i_2} - \pi_{2h_2})].\frac{1}{2}$. In the main text Fig. 6 shows the fixation probability $\rho_{A_1^1A_1^2,\, A_1^1A_2^2}$ (both Method I and Method II) with respect to varying selection intensities in the $A_1^1A_1^2$, $A_1^1A_2^2$ edge of the

tetrahedron simplex that contains the multiple game dynamics for a combination of two games with two strategies each. While this is the general case where both the games matter, Fig. A.3 is a particular case where the payoff in game $A^1$ is zero. Here, there is no difference between Method II and SGD. However, in Method I, its results differ from SGD. Eq. A.40 becomes,

$$\begin{aligned} &\mid (\frac{1}{Z} + \frac{w}{Z^2}[\sum_{m=1}^{Z-1}\sum_{\gamma=1}^{m}(\pi_{21} - \pi_{22})]) - (\frac{1}{Z} + \frac{w}{2Z^2}[\sum_{m=1}^{Z-1}\sum_{\gamma=1}^{m}(\pi_{21} - \pi_{22})]) \mid \\ &= \mid (\frac{w}{Z^2}[\sum_{m=1}^{Z-1}\sum_{\gamma=1}^{m}(\pi_{21} - \pi_{22})].\frac{1}{2}) \mid . \end{aligned} \tag{A.41}$$

Thus the kind of mapping method that one chooses becomes important in multi game dynamics as there are various ways of mapping payoffs to fitness especially when we remove the assumption that the selection intensity are the same value $w$ for all $N$ games i.e. the value $w_j$ would be different from one game $j$ to another.

# References


[1] Hofbauer J, Sigmund K. Evolutionary Games and Population Dynamics. Cambridge, UK: Cambridge University Press; 1998.

[2] Nowak MA. Evolutionary dynamics. Cambridge MA: Harvard University Press; 2006.

[3] Gokhale CS, Traulsen A. Evolutionary multiplayer games. Dynamic Games and Applications. 2014;4:468–488.

[4] Gokhale CS, Traulsen A. Evolutionary games in the multiverse. Proceedings of the National Academy of Sciences USA. 2010;107:5500–5504.

[5] Cressman R, Gaunersdorfer A, Wen JF. Evolutionary and dynamic stability in symmetric evolutionary games with two independent decisions. International Game Theory Review. 2000;2(1).

[6] Chamberland M, Cressman R. An example of dynamic (in) consistency in symmetric extensive form evolutionary games. Games and Economic Behavior. 2000;30:319–326.

[7] Hashimoto K. Unpredictability induced by unfocused games in evolutionary game dynamics. Journal of Theoretical Biology. 2006;241(3):669–675.

[8] Hilbe C, Sigmund K. Incentives and opportunism: from the carrot to the stick. Proceedings of the Royal Society B. 2010;277:2427–2433.

[9] Gaunersdorfer A, Hofbauer J, Sigmund K. The dynamics of asymmetric games. Theoretical Population Biology. 1991;29:345–357.

[10] Traulsen A, Hauert C. Stochastic evolutionary game dynamics. In: Schuster HG, editor. Reviews of Nonlinear Dynamics and Complexity. vol. II. Weinheim: Wiley-VCH; 2009. p. 25–61.

[11] Hauert C, Traulsen A, Brandt H, Nowak MA, Sigmund K. Via freedom to coercion: the emergence of costly punishment. Science. 2007;316:1905–1907.

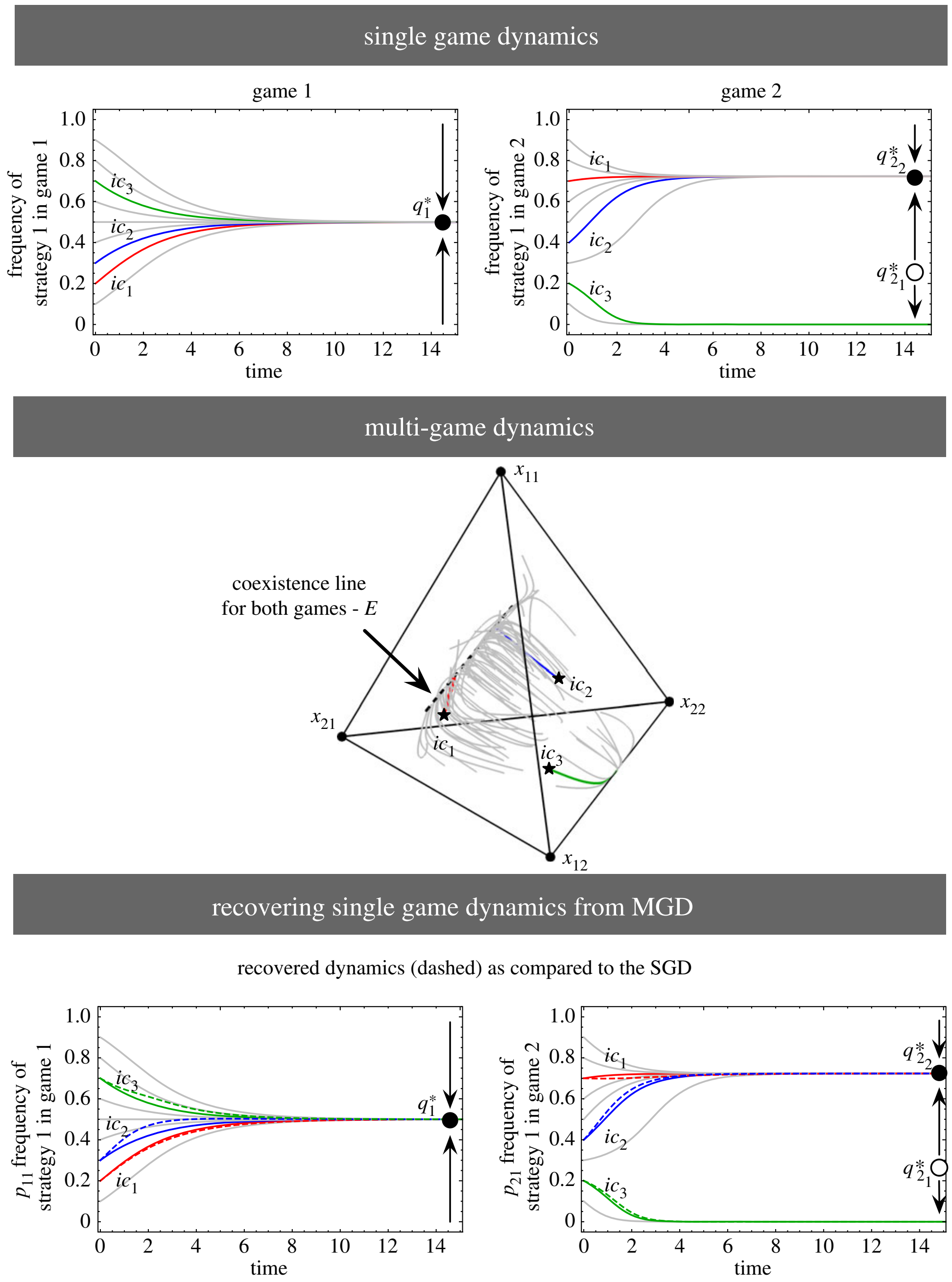


**Figure A.4.** Two games with two strategies. The SGD of a 2-player and a 3-player game from equations (3.1) are shown in the top panel. Initial conditions of the highlighted trajectories correspond to the ones used in the MGD. The vertices of an $S_4$ simplex (tetrahedron) denote these 'categorical strategies'. The asterisks depict the initial conditions ($ic_1$, $ic_2$, and $ic_3$) chosen to correspond to the initial conditions from the SGD. Other random initial conditions are plotted in grey. Recovering the SGDs from the MGD, we see that $p_{11}$ (playing strategy 1 in game 1, dashed lines) converges to $q_1^* = 0.5$ which is the equilibrium solution for strategy 1 in game 1. If we start above the unstable equilibrium solution for game 2, i.e. $q_{2_1}^* = 0.27$, then $p_{21}$ (playing strategy 1 in game 2, dashed lines) converges to $q_{2_2}^* = 0.73$, the stable equilibrium solution. For trajectories commencing below the unstable equilibrium, strategy 1 goes extinct. Comparing the recovered (dashed) dynamics to the SGD (solid), we see that while the equilibria of the recovered dynamics are the same as that of the SGD, the trajectories do not follow the same path. This is because the trajectories traverse a higher dimension which offers optional paths to the same equilibrium solutions. The initials conditions for ($x_{11}$, $x_{12}$, $x_{21}$, $x_{22}$) used in these plots are: $ic_1 = (0.1, 0.1, 0.6, 0.2)$, $ic_2 = (0.2, 0.1, 0.2, 0.5)$, and $ic_3 = (0.1, 0.6, 0.1, 0.2)$. (Online version in colour.)

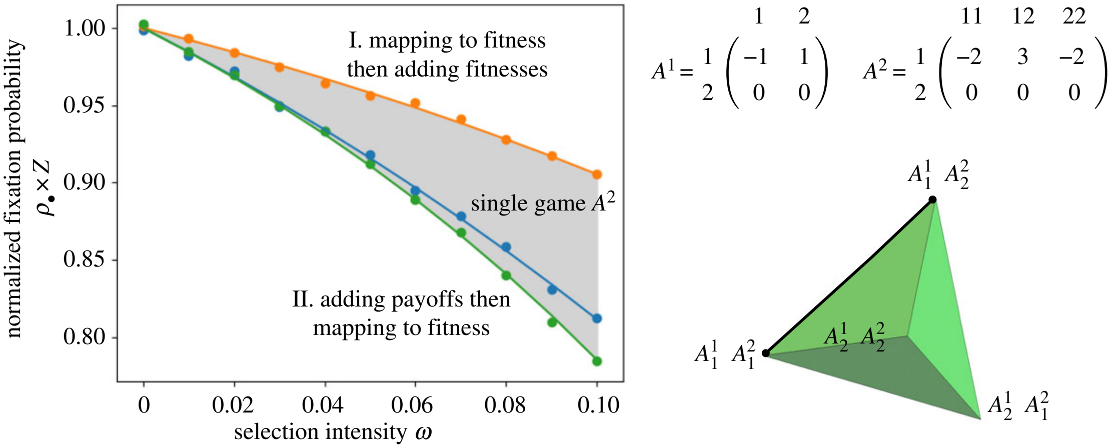


**Figure A.5.** Fixation probability of a single individual playing $A_1^1A_1^2$ strategy on the edge $A_1^1A_1^2 \rightleftharpoons A_1^1A_2^2$, i.e. $\rho_{A_1^1A_1^2,A_1^1A_2^2}$ varying with selection intensity for a combination of two games having two strategies each. For a population of $Z = 10$, the fixation probabilities are normalized according to the neutral fixation probability, $(1/Z) = 0.1$. We look at the edge $A_1^1A_1^2 \rightleftharpoons A_1^1A_2^2$ where $A^1$ is the same for both vertices, i.e. neutral in both the vertices, and $A^2$ is what matters. Since the payoff of playing strategy 2 in $A^2$ is greater than playing strategy 1 ($\pi_{22} > \pi_{21}$), the fixation probability decreases (see the electronic supplementary material for more details). The line labelled 'single game' corresponds to $A^2$. The plots from Method I (mapping payoffs to fitnesses and then adding the fitnesses to get the combined fitness) and Method II (adding the payoffs first, and then performing the payoff to fitness mapping) for a combination of the two games $A^1$ and $A^2$ show how the MGD is different from the SGD. Adding another game to $A^2$ modifies the dynamics. Within the MGD, the two methods of mapping from payoffs to fitness, i.e. Methods I and II show different results. The shaded region (calculated in the electronic supplementary material) shows this difference between the two methods with increasing selection intensity. The results from analytics and stochastic simulations are plotted as solid lines and symbols, respectively. The simulations are averaged over $10^6$ realizations. (Online version in colour.)
